# Negative attitude and low intention to vaccinate universally against varicella among public health professionals and parents in the Netherlands: two internet surveys

**DOI:** 10.1186/s12879-016-1442-1

**Published:** 2016-03-15

**Authors:** Alies van Lier, Alma Tostmann, Irene A. Harmsen, Hester E. de Melker, Jeannine L. A. Hautvast, Wilhelmina L. M. Ruijs

**Affiliations:** Centre for Infectious Disease Control, National Institute for Public Health and the Environment (RIVM), P.O. Box 1 (internal P.O. Box 75), 3720 BA Bilthoven, The Netherlands; Regional Public Health Service ‘GGD Gelderland-Zuid’, P.O. Box 1120, 6501 BC Nijmegen, The Netherlands; Department of Work & Social Psychology, Maastricht University, P.O. Box 616, 6200 MD Maastricht, The Netherlands; Academic Collaborative Centre AMPHI, Department of Primary and Community Care, Radboud university medical center, P.O. Box 9101, 6500 HB Nijmegen, The Netherlands

**Keywords:** Varicella Zoster Virus, Varicella, Chickenpox, Vaccination, Epidemiology, Intention

## Abstract

**Background:**

Prior to introduction of universal varicella vaccination, it is crucial to gain insight into the willingness to vaccinate among the population. This is because suboptimal national vaccination coverage might increase the age of infection in children, which will lead to higher complication rates. We studied the attitude and intention to vaccinate against varicella among Dutch public health professionals who execute the National Immunisation Programme (NIP), and parents.

**Methods:**

Medical doctors and nurses of regional public health services (RPHS) and child health clinics (CHC), and a random sample of parents received an internet survey on varicella vaccination. Separate logistic regression models were used to identify determinants for a positive attitude (professionals) or a positive intention (parents) to vaccinate against varicella within the NIP (free of charge).

**Results:**

The questionnaire was completed by 181 RPHS professionals (67 %), 260 CHC professionals (46 %), and 491 parents (33 %). Of professionals, 21 % had a positive attitude towards universal varicella vaccination, while 72 % preferred to limit vaccination to high-risk groups only. Of parents, 28 % had a positive intention to vaccinate their child against varicella within the NIP. The strongest determinant for a positive attitude or intention to vaccinate against varicella among professionals and parents was the belief that varicella is a disease serious enough to vaccinate against.

**Conclusions:**

We showed that a majority of the Dutch public health professionals and parents in this study have a negative attitude or low intention to vaccinate universally against varicella, as a result of the perceived low severity of the disease. Most participating professionals support selective vaccination to prevent varicella among high-risk groups.

**Electronic supplementary material:**

The online version of this article (doi:10.1186/s12879-016-1442-1) contains supplementary material, which is available to authorized users.

## Background

The Dutch National Immunisation Programme (NIP) is a voluntary prevention programme that is offered free of charge by the government since 1957. Vaccinations are administered by public health professionals of child health clinics (CHC; children <4 years) and youth health departments of regional public health services (RPHS; school-aged children). At this moment, the NIP includes vaccination against twelve diseases: diphtheria, pertussis, tetanus, poliomyelitis, *Haemophilus influenzae* serotype b (Hib), hepatitis B, pneumococcal disease (10 serotypes), measles, mumps, rubella, meningococcal C disease, and human papilloma virus infection (2 serotypes). Vaccination coverage in the Netherlands has been high for many decades (≥95 % for newborns) [1], except for HPV (50–60 %). The Dutch Health Council advises whether newly available vaccines should be incorporated in the NIP, using an assessment framework of seven criteria [2]. These criteria cover the seriousness and extent of the disease burden, the effectiveness and safety of the vaccination, the acceptability of the vaccination, the efficiency of the vaccination, and the priority of the vaccination. Varicella vaccine is one of the vaccines currently under consideration by the Health Council, because it could lower varicella disease burden [3]. The varicella vaccine can be administered separately or as combination vaccine MMRV (measles-mumps-rubella-varicella), the latter is known to give a higher risk on febrile seizures. A second dose is advised to prevent breakthrough varicella [4]. In theory, with a prescription from a medical doctor, Dutch parents could purchase the varicella vaccine themselves to be administered by their own general practitioner. In practice this is done only to a very limited extent: for children below 5 years of age ~165 varicella vaccines were delivered by Dutch community pharmacies in 2014 (source: Foundation for Pharmaceutical Statistics (SFK)).

From a public health perspective, reaching high vaccination coverage is essential for successful implementation of varicella vaccination. A vaccination coverage >94 % is needed to achieve herd immunity to eliminate endemic transmission in the Netherlands [5] and to prevent the formation of an unvaccinated population at risk of delayed infection at older age and hence more severe disease [3, 6]. However, such high vaccination coverage may not be feasible in practice. Another concern about varicella vaccination at high vaccination coverage is the initial potential rise in herpes zoster incidence that might be caused by diminishing exogenous boosting in latently infected individuals when varicella incidence decreases (Hope-Simpsons’ hypothesis) [7, 8], a burden which can be partly mitigated by herpes zoster vaccination in elderly [9]. In the long run, herpes zoster incidence is expected to decrease because reactivation after vaccination is expected to be rarer than after natural infection [10].

An alternative approach to universal vaccination would be to vaccinate high-risk groups to prevent severe varicella as is done in some European countries [11]. High-risk groups include certain immunocompromised people without previous infection and susceptible adolescents. Because the live (attenuated) varicella vaccine is contraindicated in people with severe immunodeficiency or acquired immunosuppression caused by disease or treatment, or during pregnancy, the vaccine cannot always be given. Vaccination of contacts without varicella history (healthcare professionals or family members) or vaccination prior to immunosuppressive treatment is then advised. With this selective vaccination approach, aimed at individual rather than community protection, varicella zoster virus (VZV) transmission and circulation will not be affected given the small number of eligible people.

Notwithstanding that severe complications do occur, the generally mild course of varicella, might give rise to reluctance regarding varicella vaccination among the Dutch population. In the Netherlands, varicella is usually contracted at young age and the hospitalisation rate is relatively low [12, 13]. A previous survey showed that 54 % of Dutch parents were unwilling to vaccinate their child against varicella [14]. Public health professionals play a crucial role in the implementation of the NIP [15]. As they inform parents about the importance of vaccination and deal with their critical questions, they would need to support varicella vaccination, if it were to be introduced. Given the perceived increased risks in case of suboptimal vaccination coverage, we investigated the attitude and intention to vaccinate against varicella and associated determinants among public health professionals who execute the NIP, and parents in the Netherlands.

## Methods

### Study population and design

#### Public health professionals

The following professionals, involved in the execution of the NIP, were invited to participate in an internet survey:

- Medical doctors and nurses (*N =* 269) of infectious disease control departments of all 28 RPHS in the Netherlands;
- Medical doctors and nurses (*N =* 563) of 8 regional organisations executing CHC in the eastern part of the Netherlands, covering about 15–20 % of the Dutch population.

Invitations and reminders were sent in the spring and autumn of 2012. Professionals who participated gave their informed consent by filling out the questionnaire. The questionnaire contained questions on several background characteristics, knowledge about VZV, attitude towards universal varicella vaccination, and beliefs about the disease varicella and varicella vaccination. Most questions were formulated as statements on which the level of agreement was measured using a 5-point Likert scale.

#### Parents

Fifteen hundred parents with ≥1 child aged between 0–4 years were randomly selected from the national immunisation register Præventis [16], after approval by its registration committee. This register includes all Dutch children (it has a link to the national population register). In November 2012, parents received an invitation letter from the RIVM with a link to an internet survey, followed by a reminder letter after three weeks to those parents who had not yet participated. Parents who participated gave their informed consent by filling out the questionnaire. The questionnaire for parents was, where possible, similar to that of professionals (Additional file 1), which provided the opportunity to compare knowledge and beliefs of professionals and parents. Comparison has the potential to learn if education and training on VZV may enhance acceptance of varicella vaccination.

According to Dutch law (i.e., the Medical Research Involving Human Subjects Act (WMO)), the nature of these general internet-based surveys among healthy volunteers does not require formal medical ethical approval (www.ccmo.nl).

### Data analysis

#### Attitude and intention to vaccinate against varicella

The primary outcome for professionals was their attitude towards universal varicella vaccination. Professionals were divided into two groups: a) positive attitude (=universal varicella vaccination for all children) and b) negative attitude (nobody or selected groups should be offered varicella vaccination or no answer). The primary outcome for parents was the intention to vaccinate their child against varicella if a vaccine were available through the NIP – divided into a) positive intention (=‘yes, definitely’ or ‘probably yes’), b) neutral (=‘neutral’), and c) negative (=‘no, never’ or ‘probably not’).

#### Determinants of attitude and intention to vaccinate against varicella

##### Knowledge about VZV

A knowledge score was calculated based on five different knowledge questions, with a maximum of 5 points (1 point per correct answer, Additional file 1). Respondents were then scored as having ‘limited knowledge’ (0–2 points), ‘moderate knowledge’ (3 points) or ‘good knowledge’ (4–5 points) about VZV. Differences in knowledge between professionals and parents, and between medical doctors and nurses were tested using Pearson’s χ^2^ or Fisher’s exact test. To correct for multiple testing, the Benjamini-Hochberg method was applied with a false discovery rate of 0.05 [17].

##### Beliefs about the disease varicella and varicella vaccination

To get insight into the perceived severity of varicella, respondents were asked to rank the seriousness of different vaccine preventable diseases, and varicella. Beliefs about varicella and varicella vaccination were measured by 7 statements (Additional file 1). To study differences in beliefs between medical doctors, nurses and parents, mean scores and associated simultaneous Bonferroni confidence intervals with overall coverage of at least 95 % were calculated.

##### Logistic regression analyses

For professionals and parents, separate logistic regression models were used to identify determinants of a positive attitude (professionals) or positive intention (parents) to vaccinate universally against varicella (free of charge). For parents, respondents with a neutral intention and respondents with a negative intention were merged into one group. The following potential determinants were included in the univariable and multivariable logistic regression analyses: sex, age, profession, education level, VZV knowledge score, and beliefs about the disease varicella and varicella vaccination (see Additional file 1 for more details on included questionnaire items). For these regression analyses, the agreement on statements regarding beliefs about the disease varicella and varicella vaccination was divided into three categories: a) no agreement (‘strongly disagree’ or ‘disagree’), b) neutral (‘neutral’), and c) agreement (‘agree’ or ‘strongly agree’). Crude and adjusted odds ratios (OR) and 95 % confidence intervals (CI) were calculated based on cases without missing answers (professionals *N =* 404, parents *N =* 491). A determinant was considered to be statistically significantly associated with the outcome if the *P* value was <0.05. The logistic regression analyses were conducted in SAS 9.3, descriptive analyses in SPSS 19.0.

## Results

### Response and background characteristics

The questionnaire was completed by 181 RPHS professionals (67 % response), 260 CHC professionals (46 % response), and 491 parents (33 % response). Background characteristics are presented in Table 1. The majority of respondents were women. A small proportion of parents had a low education (10 %) or low income (4 %) and for the majority of the respondents both parents were born in the Netherlands (86 %).

**Table 1:** Background characteristics of public health professionals and parents

	Professionals (RPHS & CHC combined)						Parents	
	Medical doctors (*N =* 162)		Nurses (*N =* 279)		Total (*N =* 441)		(*N =* 491)	
Background characteristics	N	%	N	%	N	%	N	%
Sex								
Male	37	22.8	13	4.7	50	11.3	90	18.3
Female	125	77.2	266	95.3	391	88.7	401	81.7
Age								
Younger than 30 years	8	5.0	30	10.9	38	8.7	94	19.1
30–34 years	16	9.9	32	11.6	48	11.0	189	38.5
35–39 years	16	9.9	26	9.4	42	9.6	148	30.1
40 years or older	121	75.2	188	68.1	309	70.7	60	12.2
Organisation								
Regional Public Health Service (RPHS)	74	45.7	107	38.4	181	41.0	-	-
Child Health Clinic (CHC)	88	54.3	172	61.6	260	59.0	-	-
Education^c^								
Low	n.a.	n.a.	n.a.	n.a.	n.a.	n.a.	51	10.4
Middle	n.a.	n.a.	n.a.	n.a.	n.a.	n.a.	166	33.8
High	n.a.	n.a.	n.a.	n.a.	n.a.	n.a.	274	55.8
Ethnicity								
Both parents born in the Netherlands	n.a.	n.a.	n.a.	n.a.	n.a.	n.a.	423	86.3
At least one parent born in another country	n.a.	n.a.	n.a.	n.a.	n.a.	n.a.	67	13.7
Net monthly household income								
Low (≤ €1150)	n.a.	n.a.	n.a.	n.a.	n.a.	n.a.	18	4.4
Middle (€1151–€3050)	n.a.	n.a.	n.a.	n.a.	n.a.	n.a.	191	47.2
High (≥ €3051)	n.a.	n.a.	n.a.	n.a.	n.a.	n.a.	196	48.4
No answer	n.a.	n.a.	n.a.	n.a.	n.a.	n.a.	86	
Household size								
< 4 persons	n.a.	n.a.	n.a.	n.a.	n.a.	n.a.	163	33.2
4 persons	n.a.	n.a.	n.a.	n.a.	n.a.	n.a.	228	46.4
> 4 persons	n.a.	n.a.	n.a.	n.a.	n.a.	n.a.	100	20.4
Number of children								
None	31	19.1	73	26.2	104	23.6	-	-
One child	15	9.3	30	10.8	45	10.2	160	32.6
Two children	48	29.6	104	37.3	152	34.5	234	47.7
More than two children	68	42.0	72	25.8	140	31.7	97	19.8
Participation of child(ren) in NIP?								
Yes, fully immunised	124	96.1	185	90.7	309	92.8	465	94.7
Yes, partially immunised	4	3.1	19	9.3	23	6.9	13	2.6
No, not immunised	1	0.8	0	0.0	1	0.3	10	2.0
Don’t know	0	0.0	0	0.0	0	0.0	3	0.6
Change in opinion on vaccination?^a^								
No	126	77.8	231	83.1	357	81.1	420	85.5
Yes, more inclined	14	8.6	16	5.8	30	6.8	9	1.8
Yes, less inclined	11	6.8	14	5.0	25	5.7	47	9.6
Don’t know	11	6.8	17	6.1	28	6.4	15	3.1
Influence on opinion on vaccination?								
Anthroposophical philosophy	1	0.6	7	2.6	8	1.9	8	1.6^b^
Homeopathic philosophy	0	0.0	3	1.1	3	0.7	16	3.3^b^
Alternative medicine	0	0.0	2	0.7	2	0.5	10	2.0^b^
Religion	1	0.6	3	1.1	4	0.9	26	5.3^b^
Other	13	8.3	19	6.9	32	7.4	29	5.9^b^
None of the above	141	90.4	240	87.6	381	88.6	417	84.9^b^

RPHS = regional public health service; CHC = child health clinic

^a^With respect to the five years preceding the survey

^b^Percentages add up to more than 100 % because a respondent can be within multiple categories

^c^Low: no, primary, lower vocational or lower secondary education; middle: secondary vocational or higher secondary education; high: higher professional or university education

Full immunisation of their own children according to the NIP was reported by 93 % of professionals with children and by 95 % of parents. Over the past five years, 6 % of professionals and 10 % of parents had become less willing to vaccinate. The majority of the respondents (professionals 89 %, parents 85 %) reported that their vaccination beliefs were not influenced by e.g., anthroposophical philosophy, homeopathic philosophy, alternative medicine or religious ideas.

### Attitude and intention to vaccinate against varicella

Only 21 % of professionals (medical doctors 28 %, nurses 17 %) had a positive attitude towards varicella vaccination and responded that varicella vaccination should be offered to all infants, mainly to prevent severe cases and complications (Table 2). The majority of professionals (72 %) believed that only select groups should be eligible for varicella vaccination, and 7 % responded that nobody should be vaccinated against varicella. Among professionals who supported selective vaccination, 74 % mentioned high-risk groups for severe varicella, 9 % all 9-year-olds without varicella history, and 16 % both.

**Table 2:** Attitude to vaccinate against varicella among professionals

	Professionals (RPHS & CHC combined)					
	Medical doctors (*N =* 162)		Nurses (*N =* 279)		Total (*N =* 441)	
Attitude towards universal varicella vaccination	N	%	N	%	N	%
All infants	45	28.0	46	17.0	91	21.1
Select groups	108	67.1	204	75.3	312	72.2
*- Risk groups (high risk of severe course)*	*76*	*70.4*	*156*	*76.5*	*232*	*74.4*
*- All susceptible 9-year olds*	*12*	*11.1*	*17*	*8.3*	*29*	*9.3*
*- Both*	*20*	*18.5*	*31*	*15.2*	*51*	*16.3*
Nobody	8	5.0	21	7.7	29	6.7
Missing answer	1		8		9	

RPHS = regional public health service; CHC = child health clinic

**Table 3:** Intention to vaccinate against varicella among parents

	Parents (*N =* 491)	
Intention regarding vaccination of own child against varicella within the NIP (free of charge)	N	%
Yes, definitely	42	8.6
Probably yes	96	19.6
Neutral	101	20.6
Probably not	152	31.0
No, never	100	20.4

Among parents, 28 % had a positive intention to vaccinate their child against varicella offered within the NIP (free of charge); 21 % were indecisive, and 51 % had a negative intention (Table 3). If parents were to be charged for the vaccination, the positive intention dropped to 20 %.

### Determinants of attitude and intention to vaccinate against varicella

#### Knowledge about VZV

Based on the knowledge score, most respondents had a ‘moderate’ or ‘good knowledge’ about VZV in general (professionals 86 %, parents 64 %) (Table 4). However, the relation of varicella with herpes zoster was largely unknown (professionals 44 %, parents 6 %). Furthermore, approximately half of the professional respondents did not know how often varicella related events occur, such as general practitioner consultation and hospitalisation.

**Table 4:** Knowledge of public health professionals and parents about varicella zoster virus (VZV)

	Professionals (RPHS & CHC combined)							Parents		
	Medical doctors (*N =* 162)		Nurses (*N =* 279)		Total (*N =* 441)			(*N =* 491)		
Questionnaire item	N	%	N	%	N	%	*p*-value*^,^**	N	%	*p*-value*^,^***
If you never have had varicella, you can not get herpes zoster^a^										
Right *(correct answer)*	102	63.4	91	32.7	193	44.0	**<0.0001**	30	6.1	**<0.0001**
Wrong	50	31.1	124	44.6	174	39.6		218	44.4	
Don’t know	9	5.6	63	22.7	72	16.4		243	49.5	
If the blisters have dried up, you are no longer infectious^a^										
Right *(correct answer)*	155	95.7	264	95.3	419	95.4	1.000	430	87.6	**<0.0001**
Wrong	6	3.7	9	3.2	15	3.4		21	4.3	
Don’t know	1	0.6	4	1.4	5	1.1		40	8.1	
In general, you will get varicella only once in your life^a^										
Right *(correct answer)*	145	90.1	220	79.4	365	83.3	**0.004**	401	81.7	0.546
Wrong	14	8.7	55	19.9	69	15.8		69	14.1	
Don’t know	2	1.2	2	0.7	4	0.9		21	4.3	
What percentage of the Dutch population has had varicella before the age of 12 years?^a^										
50 %	3	1.9	15	5.4	18	4.1		92	18.7	
75 %	42	26.1	129	46.6	171	39.0		252	51.3	
95 % *(correct answer)*	114	70.8	133	48.0	247	56.4	**<0.0001**	147	29.9	**<0.0001**
100 %	2	1.2	0	0.0	2	0.5		0	0.0	
How many people visit their general practitioner for varicella in the Netherlands each year?										
1 in 5	4	2.5	11	4.0	15	3.4		66	13.4	
1 in 50	20	12.6	68	24.5	88	20.1		160	32.6	
1 in 500 *(correct answer)*	76	47.8	128	46.0	204	46.7	0.765	175	35.6	**0.001**
1 in 5000	59	37.1	71	25.5	130	29.7		90	18.3	
How many people are being hospitalised for varicella or its complications in the Netherlands each year?										
1 in 50	5	3.1	54	19.5	59	13.5		89	18.1	
1 in 500	7	4.3	23	8.3	30	6.8		77	15.7	
1 in 5000	27	16.8	59	21.3	86	19.6		139	28.3	
1 in 50000 *(correct answer)*	122	75.8	141	50.9	263	60.0	**<0.0001**	186	37.9	**<0.0001**
How many people die due to varicella or its complications in the Netherlands each year?^a^										
Less than 10 *(correct answer)*	142	88.2	237	85.6	379	86.5	0.471	344	70.1	**<0.0001**
10–100	17	10.6	33	11.9	50	11.4		129	26.3	
100–1000	1	0.6	2	0.7	3	0.7		13	2.6	
More than 1000	1	0.6	5	1.8	6	1.4		5	1.0	
Knowledge score VZV**** (max. 5 points)							**<0.0001**			**<0.0001**
Limited knowledge (0–2 points)	6	3.8	55	20.3	61	14.2		177	36.0	
Moderate knowledge (3 points)	31	19.6	90	33.2	121	28.2		209	42.6	
Good knowledge (4–5 points)	121	76.6	126	46.5	247	57.6		105	21.4	

RPHS = regional public health service; CHC = child health clinic

*Pearson’s χ^2^ or Fisher’s exact test; *p*-values in bold indicate results that are considered statistically significant after correction for multiple testing by the Benjamini–Hochberg method at a false discovery rate of 0.05

**P-value for comparison of the proportion with a correct answer (with the exception of the knowledge score) between medical doctors and nurses

***P-value for comparison of the proportion with a correct answer (with the exception of the knowledge score) between professionals and parents

****Knowledge score VZV: sum of the 5 items above with an ^a^ where each correct answer was awarded with 1 point, a wrong or missing answer with 0 points

For most of the knowledge items – including total knowledge score – knowledge of professionals was better than that of parents, and medical doctors had a better knowledge than nurses (Table 4).

#### Beliefs about the disease varicella and varicella vaccination

**Fig. 1:**
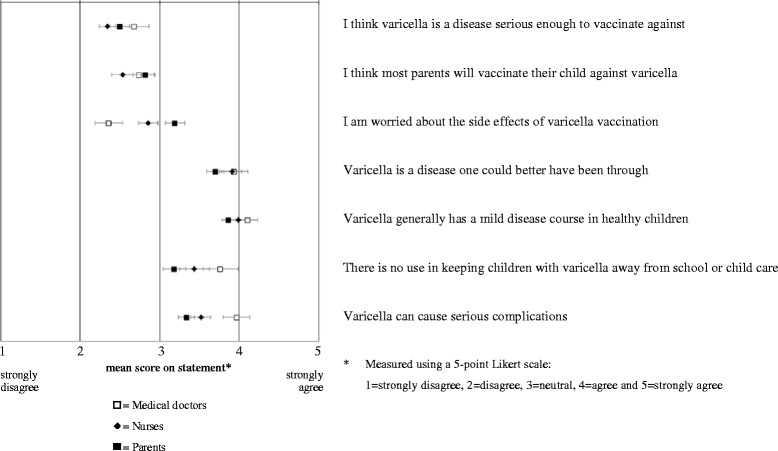
Mean score (and associated simultaneous Bonferroni confidence intervals with overall coverage of at least 95 %) on statements regarding the beliefs about the disease varicella and varicella vaccination

Based on the mean ranking score for the seriousness of tetanus, poliomyelitis, pertussis, measles, mumps, rubella, pneumococcal disease, meningococcal disease, and varicella, it was clear that both professionals and parents consider varicella to be the mildest of these diseases. Most respondents perceived varicella as ‘a disease one could better have been through’ (professionals 77 %, parents 65 %) and responded that varicella ‘generally has a mild disease course in healthy children’ (professionals 91 %, parents 80 %). However, 66 % of professionals and 42 % of parents agreed that varicella is ‘able to cause serious complications’. Only 11 % of professionals and 20 % of parents indicated that varicella is ‘a disease serious enough to vaccinate against’ but despite this, 21 % of professionals, and 25 % of parents expected that ‘most parents will vaccinate their child against varicella’. Figure 1 shows that beliefs about the disease varicella and varicella vaccination among professionals and parents were in general quite similar.

#### Logistic regression analyses

Among professionals, the following determinants were statistically significantly associated with a positive attitude towards universal varicella vaccination in the multivariable logistic regression analysis (Table 5): the beliefs that ‘varicella is a disease serious enough to vaccinate against’ (positively), ‘most parents will vaccinate their child against varicella’ (positively), and ‘varicella generally has a mild disease course in healthy children’ (negatively).

**Table 5:** Determinants for a positive attitude (professionals) or positive intention (parents) regarding universal varicella vaccination from univariable and multivariable logistic regression analysis

	Professionals (RPHS & CHC combined)				Parents			
Potential determinants	N	Outcome^a^: positive attitude % [95 % CI]	Crude OR [95 % CI]	Adjusted OR [95 % CI]	N	Outcome^a^: positive intention % [95 % CI]	Crude OR [95 % CI]	Adjusted OR [95 % CI]
Sex								
Male	40	22.5 [12.3–37.5]	Reference	Reference	90	27.8 [19.6–37.8]	Reference	Reference
Female	364	20.6 [16.8–25.1]	0.9 [0.4–2.0]	1.7 [0.6–4.6]	401	28.2 [24.0–32.8]	1.0 [0.6–1.7]	1.0 [0.5–2.0]
Age								
Younger than 30 years	38	28.9 [17.0–44.8]	Reference	Reference	94	23.4 [16.0–32.9]	Reference	Reference
30–34 years	48	29.2 [18.2–43.2]	1.0 [0.4–2.6]	0.7 [0.2–2.2]	189	35.4 [29.0–42.5]	1.8 [1.0–3.2]^b^	1.8 [0.8–3.8]
35–39 years	40	25.0 [14.2–40.2]	0.8 [0.3–2.2]	0.8 [0.2–2.9]	148	20.3 [14.6–27.5]	0.8 [0.4–1.6]	1.1 [0.5–2.6]
40 years or older	278	17.6 [13.6–22.5]	0.5 [0.2–1.1]	0.4 [0.1–1.0]	60	31.7 [21.3–44.2]	1.5 [0.7–3.1]	1.2 [0.4–3.4]
Profession								
Medical doctor	151	27.8 [21.3–35.4]	Reference	Reference	-			
Nurse	253	16.6 [12.5–21.7]	0.5 [0.3–0.8]^b^	1.0 [0.5–1.9]	-			
Education level								
High					274	27.0 [22.1–32.6]	Reference	Reference
Medium					166	27.1 [20.9–34.3]	1.0 [0.7–1.6]	0.9 [0.5–1.7]
Low					51	37.3 [25.3–51.0]	1.6 [0.9–3.0]	1.0 [0.4–2.4]
Knowledge about VZV (max. 5 points)								
Limited knowledge (0–2 points)	56	17.9 [10.0–29.8]	Reference	Reference	177	35.0 [28.4–42.3]	Reference	Reference
Moderate knowledge (3 points)	113	18.6 [12.5–26.7]	1.1 [0.5–2.4]	1.0 [0.4–2.9]	209	23.0 [17.8–29.1]	0.6 [0.4–0.9]^b^	0.9 [0.5–1.6]
Good knowledge (4–5 points)	235	22.6 [17.7–28.3]	1.3 [0.6–2.8]	1.1 [0.4–2.8]	105	26.7 [19.1–35.8]	0.7 [0.4–1.1]	1.4 [0.7–3.0]
Beliefs about the disease varicella and varicella vaccination								
*Varicella generally has a mild disease course in healthy children*								
No agreement	12	58.3 [32.0–80.7]	Reference	Reference	22	54.5 [34.7–73.1]	Reference	Reference
Neutral	24	29.2 [14.9–49.2]	0.3 [0.1–1.3]	0.2 [0.03–1.3]	76	51.3 [40.3–62.2]	0.9 [0.3–2.3]	0.7 [0.2–2.5]
Agreement	368	19.0 [15.3–23.3]	0.2 [0.1–0.5]^b^	0.2 [0.04–0.9]^b^	393	22.1 [18.3–26.5]	0.2 [0.1–0.6]^b^	0.5 [0.1–1.4]
*Varicella can cause serious complications*								
No agreement	42	16.7 [8.3–30.6]	Reference	Reference	66	12.1 [6.3–22.1]	Reference	Reference
Neutral	98	8.2 [4.2–15.3]	0.4 [0.2–1.3]	0.6 [0.2–2.4]	218	20.2 [15.4–26.0]	1.8 [0.8–4.1]	1.0 [0.4–2.7]
Agreement	264	26.1 [21.2–31.8]	1.8 [0.8–4.2]	1.5 [0.5–4.8]	207	41.5 [35.0–48.4]	5.2 [2.3–11.3]^b^	1.3 [0.4–3.5]
*Varicella is a disease one could better have been through*								
No agreement	22	59.1 [38.7–76.7]	Reference	Reference	46	60.9 [46.5–73.6]	Reference	Reference
Neutral	73	23.3 [15.1–34.2]	0.2 [0.1–0.6]^b^	0.3 [0.1–1.3]	126	41.3 [33.1–50.0]	0.5 [0.2–0.9]^b^	0.5 [0.2–1.3]
Agreement	309	17.5 [13.6–22.1]	0.1 [0.1–0.4]^b^	0.3 [0.1–1.0]	319	18.2 [14.3–22.8]	0.1 [0.1–0.3]^b^	0.4 [0.1–0.9]^b^
*I am worried about the side effects of varicella vaccination*								
No agreement	163	30.1 [23.5–37.5]	Reference	Reference	115	27.0 [19.7–35.7]	Reference	Reference
Neutral	190	15.3 [10.8–21.1]	0.4 [0.3–0.7]^b^	0.6 [0.3–1.1]	196	29.1 [23.2–35.8]	1.1 [0.7–1.9]	0.5 [0.2–1.1]
Agreement	51	11.8 [5.5–23.4]	0.3 [0.1–0.8]^b^	0.5 [0.1–1.4]	180	27.8 [21.8–34.7]	1.0 [0.6–1.8]	0.7 [0.3–1.5]
*I think varicella is a disease serious enough to vaccinate against*								
No agreement	255	9.8 [6.7–14.1]	Reference	Reference	289	8.3 [5.6–12.1]	Reference	Reference
Neutral	107	28.0 [20.4–37.2]	3.6 [2.0–6.5]^b^	2.4 [1.2–4.7]^b^	102	32.4 [24.1–41.9]	5.3 [2.9–9.5]^b^	4.9 [2.3–10.6]^b^
Agreement	42	69.0 [54.0–80.9]	20.5 [9.5–44.5]^b^	9.9 [3.9–24.9]^b^	100	81.0 [72.2–87.5]	47.1 [24.5–90.3]^b^	26.2 [10.8–63.4]^b^
*I think most parents will vaccinate their child against varicella*								
No agreement	225	9.8 [6.5–14.4]	Reference	Reference	182	9.9 [6.3–15.1]	Reference	Reference
Neutral	96	26.0 [18.3–35.6]	3.2 [1.7–6.1]^b^	2.3 [1.1–4.9]^b^	184	24.5 [18.8–31.1]	3.0 [1.6–5.3]^b^	1.1 [0.5–2.4]
Agreement	83	44.6 [34.4–55.3]	7.4 [4.0–13.8]^b^	4.2 [2.0–8.6]^b^	125	60.0 [51.2–68.2]	13.7 [7.5–25.0]^b^	2.0 [0.8–4.6]
Total	404	20.8 [17.1–25.0]			491	28.1 [24.3–32.2]		

RPHS = regional public health service; CHC = child health clinic; OR = odds ratio; CI = confidence interval

^a^Outcome professionals: attitude towards universal childhood varicella vaccination (1 = positive (all children) or 0 = neutral or negative (nobody or high-risk groups only) attitude); outcome parents: intention regarding vaccination own child against varicella within the National Immunisation Programme (free of charge; 1 = positive or 0 = neutral or negative intention)

^b^Statistically significant (*p* < 0.05) association with the outcome

In a similar analysis among parents the beliefs that ‘varicella is a disease serious enough to vaccinate against’ (positively), and ‘varicella is a disease one could better have been through’ (negatively) were statistically significantly associated with a positive intention to vaccinate their child against varicella within the NIP (i.e., free of charge). There was no association between the knowledge score and a positive attitude (professionals) or positive intention (parents) regarding universal varicella vaccination (Table 5).

### Implementation of varicella vaccination

Among professionals, 67 % agreed that parents should have the possibility to choose between a MMR and a MMRV vaccine if vaccination is going to be introduced in the NIP, whereas 21 % did not agree and 13 % were neutral. Only 29 % of professionals had the intention to advise parents to vaccinate their children against varicella if vaccination were introduced in the NIP, and only 20 % of professionals felt able to convince parents of the importance of varicella vaccination. Furthermore, 85 % of CHC professionals expected many questions from parents about varicella vaccination, and 45 % stated that they would find it difficult to discuss the necessity of varicella vaccination with parents.

According to 36 % of parents, replacement of MMR by MMRV without the ability to choose for a separate MMR and varicella vaccine is a ‘very bad idea’ or ‘bad idea’, and 28 % of parents responded this is a ‘very good idea’ or ‘good idea’ (35 % neutral).

## Discussion

We showed that there is a negative attitude and low intention to vaccinate universally against varicella within the NIP among Dutch public health professionals and parents in this study. This negative attitude and low intention was mainly associated with the perceived low severity of varicella and the belief that it is better to experience the disease naturally. Most participating professionals (72 %) indicated that only select groups, such as high-risk groups for severe varicella or susceptible adolescents, should be eligible for varicella vaccination. Based on the knowledge score including 5 statements on VZV, most respondents had a ‘moderate’ or ‘good’ knowledge about VZV. However, as the relation of varicella with herpes zoster was largely unknown, their knowledge on crucial medical aspects may be considered limited. The attitude and intention to vaccinate universally against varicella was not associated with knowledge about VZV or education level.

Our results are in concordance with similar studies from western countries. Two studies from USA and Australia showed that parents were not convinced of the seriousness of varicella and the necessity to vaccinate [18, 19]. In a study among Quebec vaccinators, 53 % of paediatricians, 37 % of general practitioners, and 33 % of nurses considered universal vaccination of children to be useful, and vaccination of high-risk populations was favoured by the majority [20], as was also seen in our study. On the other hand, regional studies among medical professionals in Australia and the USA, showed that 50 % to 76 % had a positive attitude towards varicella vaccination, and disagreed that varicella is a benign, self-limiting disease, or normal part of childhood [21–23].

In the present study, parents seemed less positive regarding varicella vaccination than ten years ago (28 % versus 39 % [14] positive intention). Furthermore, 6 % of professionals and 10 % of parents in our study indicated that over the past five years they became less inclined to vaccinate compared to 2 % and 3 % of parents in previous studies [24]. The public debate related to vaccination against pandemic influenza A (H1N1) [25, 26], and cervical cancer also showed that vaccination is not self-evident [27]. However, introduction of universal hepatitis B vaccination went silently and high coverage was reached immediately [28]. This might be related to the high perceived severity of hepatitis B [29]. Furthermore, there was no choice between a combination vaccine with or without hepatitis B. A previous study showed that this absence of choice was not considered problematic by most parents [29]. Our study showed that absence of choice between MMR and MMRV might be a problem, as more than a third of parents were against replacement of MMR by MMRV. Among professionals two third stated that parents should have the possibility to choose between MMR and MMRV but they may be unaware of the risks of suboptimal vaccination coverage.

Previous research showed that professionals who execute the NIP play a critical role in influencing parents’ decision to vaccinate their child [30], and a strong association of vaccine-related attitudes and beliefs exists between parents and healthcare professionals in general [31]. Therefore, it is important to emphasise that only one fifth of the professionals from our study feel able to convince parents of the importance of varicella vaccination, and only a third have the intention to advise parents to vaccinate their child against varicella. So, if varicella vaccination were to be introduced, it will be a challenge to educate public health professionals to convince parents of the importance of varicella vaccination in order to reach sufficient vaccination coverage.

Benefits of universal varicella vaccination must be weighed against potential negative effects of suboptimal vaccination coverage [6, 32]. A vaccination coverage >94 % is needed to achieve herd immunity for varicella in the Netherlands. However, only 28 % of parents in our study indicated a positive intention to vaccinate against varicella. A higher mean age of infection due to suboptimal vaccination coverage would come with an increased complication rate. In addition, a potential initial increase in herpes zoster incidence at high vaccination coverage means that the decision whether or not to introduce varicella vaccination must be taken with caution. If varicella vaccination were to be introduced in the NIP, then simultaneous vaccination of elderly patients against herpes zoster should be considered, and monitoring of vaccination coverage and VZV epidemiology (including mean age of infection) would be essential. However, our results indicate that a high-risk group vaccination approach would be better accepted in the Netherlands than universal varicella vaccination.

This study has some clear limitations. The survey among CHC professionals was conducted in a specific region, and might therefore not be representative for the beliefs of all Dutch child public health professionals. However, there is no evidence for regional differences in vaccination beliefs of professionals; therefore we assume the CHC professionals sample to be a representative for the beliefs about varicella vaccination of all CHC professionals. The response rate among professionals was moderate (67 % RPHS, 46 % CHC), and the response rate of parents was rather low (33 %). The selective response of parents is demonstrated by the overrepresentation of parents with high education level (56 % versus 40 % in the total population 25–45 years [33]), and underrepresentation of parents with at least one parent born in another country (14 % versus 27 % in the total population 25–45 years [34]). Because highly educated parents have a more negative attitude towards expanding the NIP [35], our results may give an underestimation of the intention to vaccinate against varicella. This means that the overall positive intention towards varicella vaccination among parents may be more than the 28 % we found. On the other hand, our study did not show an effect of parental education level on intention to vaccinate against varicella and the NIP immunisation coverage of children of parents in our sample was comparable with national coverage. Finally, although a Dutch study showed agreement between intention and behaviour [29], other research showed that final vaccination coverage rates can be different from the intention measured in advance [36]. In our study, 21 % of parents were indecisive regarding varicella vaccination (neutral intention); in the end they may actually accept varicella vaccination. In the regression analysis, the indecisive parents were merged with parents with a negative intention. However, when we merged the indecisive parents with parents with a positive intention, the belief ‘varicella is a disease serious enough to vaccinate against’ remained the strongest determinant of a positive intention and no other determinants arised. Furthermore, linear regression analysis also showed similar results.

This study was conducted among public health doctors and nurses who are the medical professionals that execute the NIP but do not treat varicella patients themselves. It would be interesting, however, to also study the view of general practitioners and paediatricians in hospitals on universal varicella vaccination. General practitioners and paediatricians might have a better knowledge about the disease and a different opinion regarding vaccination than public health professionals, as they are the medical professionals who treat patients with varicella and have experience with its complications.

## Conclusions

To conclude, we showed that most public health professionals and parents in our study are reluctant to accept universal vaccination against varicella within the NIP, mainly because of the perceived low severity of the disease. The majority of the participating professionals do however support selective vaccination to prevent varicella among high-risk groups.

## Additional file

Additional file 1: Overview questionnaire items. (DOCX 18 kb)

## References

[1] van Lier EA, Oomen PJ, Oostenbrug MW, Zwakhals SL, Drijfhout IH, de Hoogh PA (2009). Hoge vaccinatiegraad van het Rijksvaccinatieprogramma in Nederland. [High vaccination coverage of the National Immunization Programme in the Netherlands]. Ned Tijdschr Geneeskd.

[2] Houweling H, Verweij M, Ruitenberg EJ (2010). Criteria for inclusion of vaccinations in public programmes. Vaccine.

[3] Varicella and herpes zoster vaccines (2014). WHO position paper, June 2014. Wkly Epidemiol Rec.

[4] Marin M, Broder KR, Temte JL, Snider DE, Seward JF (2010). Use of combination measles, mumps, rubella, and varicella vaccine: recommendations of the Advisory Committee on Immunization Practices (ACIP). MMWR Recomm Rep.

[5] Nardone A, de Ory F, Carton M, Cohen D, van Damme P, Davidkin I (2007). The comparative sero-epidemiology of varicella zoster virus in 11 countries in the European region. Vaccine.

[6] Rentier B, Gershon AA (2004). European Working Group on Varicella. Consensus: varicella vaccination of healthy children--a challenge for Europe. Pediatr Infect Dis J.

[7] Guzzetta G, Poletti P, Del Fava E, Ajelli M, Scalia Tomba GP, Merler S (2013). Hope-Simpson’s progressive immunity hypothesis as a possible explanation for herpes zoster incidence data. Am J Epidemiol.

[8] Hope-Simpson RE (1965). The Nature of Herpes Zoster: A Long-Term Study and a New Hypothesis. Proc R Soc Med.

[9] van Hoek AJ, Melegaro A, Gay N, Bilcke J, Edmunds WJ (2012). The cost-effectiveness of varicella and combined varicella and herpes zoster vaccination programmes in the United Kingdom. Vaccine.

[10] Heininger U, Seward JF (2006). Varicella. Lancet.

[11] Bonanni P, Breuer J, Gershon A, Gershon M, Hryniewicz W, Papaevangelou V (2009). Varicella vaccination in Europe – taking the practical approach. BMC Med.

[12] van Lier A, Smits G, Mollema L, Waaijenborg S, Berbers G, van der Klis F (2013). Varicella zoster virus infection occurs at a relatively young age in the Netherlands. Vaccine.

[13] van Lier A, van der Maas NA, Rodenburg GD, Sanders EA, de Melker HE (2011). Hospitalization due to varicella in the Netherlands. BMC Infect Dis.

[14] van de Bovenkamp-Meijer KJT, Rümke HC (2005). Twijfels over kindervaccinaties: betere voorlichting zal het draagvlak versterken. [Doubts about childhood vaccinations: improved information will enhance public support]. Medisch Contact.

[15] Simone B, Carrillo-Santisteve P, Lopalco PL. Healthcare workers role in keeping MMR vaccination uptake high in Europe: a review of evidence. Euro Surveill. 2012;17(26):pii=20206. 22790533

[16] van Lier A, Oomen P, de Hoogh P, Drijfhout I, Elsinghorst B, Kemmeren J, et al. Praeventis, the immunisation register of the Netherlands: a tool to evaluate the National Immunisation Programme. Euro Surveill. 2012;17(17):pii=20153. 10.2807/ese.17.17.20153-en 22551495

[17] Benjamini Y, Hochberg Y (1995). Controlling the false discovery rate: a practical and powerful approach to multiple testing. J Royal Statistic Society, Series B (Methodological).

[18] Taylor JA, Newman RD (2000). Parental attitudes toward varicella vaccination. The Puget Sound Pediatric Research Network. Arch Pediatr Adolesc Med.

[19] Marshall H, Ryan P, Roberton D, Beilby J (2009). Varicella immunisation practice: Implications for provision of a recommended, non-funded vaccine. J Paediatr Child Health.

[20] Boulianne NA, Duval B, Serres GD, Deceuninck G, Dionne M, Carsley J (2001). Opinions of Quebec parents and vaccinators on the usefulness of chickenpox vaccine. Can J Infect Dis.

[21] Milledge JT, Cooper CD, Woolfenden S (2003). Barriers to immunization: attitudes of general practitioners to varicella, the disease and its vaccine. J Paediatr Child Health.

[22] Schaffer SJ, Bruno S (1999). Varicella immunization practices and the factors that influence them. Arch Pediatr Adolesc Med.

[23] Newman RD, Taylor JA (1998). Reactions of pediatricians to the recommendation for universal varicella vaccination. Arch Pediatr Adolesc Med.

[24] Mollema L, Wijers N, Hahne SJ, van der Klis FR, Boshuizen HC, de Melker HE (2012). Participation in and attitude towards the national immunization program in the Netherlands: data from population-based questionnaires. BMC Public Health.

[25] Bults M, Beaujean DJ, Richardus JH, van Steenbergen JE, Voeten HA (2011). Pandemic influenza A (H1N1) vaccination in The Netherlands: parental reasoning underlying child vaccination choices. Vaccine.

[26] Bults M, Beaujean DJ, de Zwart O, Kok G, van Empelen P, van Steenbergen JE (2011). Perceived risk, anxiety, and behavioural responses of the general public during the early phase of the Influenza A (H1N1) pandemic in the Netherlands: results of three consecutive online surveys. BMC Public Health.

[27] De Melker H, Kenter G, van Rossum T, Conyn-van Spaendonck M (2012). Ontwikkelingen omtrent de HPV-vaccinatie. [Developments in HPV vaccination]. Ned Tijdschr Geneeskd.

[28] van Lier EA, Oomen PJ, Giesbers H, Conyn-van Spaendonck MAE, Drijfhout IH, Zonnenberg-Hoff IF, et al. Vaccinatiegraad Rijksvaccinatieprogramma Nederland; verslagjaar 2014. [Immunisation coverage National Immunisation Programme in the Netherlands; year of report 2014]. Bilthoven: National Institute for Public Health and the Environment (RIVM); 2014 (RIVM report 150202003). Dutch.

[29] Harmsen IA, Lambooij MS, Ruiter RA, Mollema L, Veldwijk J, van Weert YJ (2012). Psychosocial determinants of parents’ intention to vaccinate their newborn child against hepatitis B. Vaccine.

[30] Smith PJ, Kennedy AM, Wooten K, Gust DA, Pickering LK (2006). Association between health care providers’ influence on parents who have concerns about vaccine safety and vaccination coverage. Pediatrics.

[31] Mergler MJ, Omer SB, Pan WK, Navar-Boggan AM, Orenstein W, Marcuse EK (2013). Association of vaccine-related attitudes and beliefs between parents and health care providers. Vaccine.

[32] Brisson M, Edmunds WJ, Gay NJ, Law B, De Serres G (2000). Modelling the impact of immunization on the epidemiology of varicella zoster virus. Epidemiol Infect.

[33] Statistics Netherlands. Education level of the Dutch population in 2013. Voorburg: CBS; 2013; Available from: http://statline.cbs.nl/Statweb/publication/?DM=SLNL&PA=71738ned&D1=0&D2=0&D3=0,2-3&D4=1-4&D5=54&HDR=T&STB=G4,G1,G2,G3&VW=T. Accessed at 17 July 2014.

[34] Statistics Netherlands. Ethnicity of the Dutch population in 2013. Voorburg: CBS; 2013; Available from: http://statline.cbs.nl/StatWeb/publication/?DM=SLNL&PA=37325&D1=0&D2=0&D3=0,106-109&D4=0&D5=0-2&D6=17&HDR=T&STB=G1,G2,G3,G4,G5&VW=T. Accessed at 17 July 2014.

[35] Hak E, Schonbeck Y, De Melker H, Van Essen GA, Sanders EA (2005). Negative attitude of highly educated parents and health care workers towards future vaccinations in the Dutch childhood vaccination program. Vaccine.

[36] Kwon Y, Cho HY, Lee YK, Bae GR, Lee SG (2010). Relationship between intention of novel influenza A (H1N1) vaccination and vaccination coverage rate. Vaccine.

