# Supplementary material for: Negative attitude and low intention to vaccinate universally against varicella among public health professionals and parents in the Netherlands: two internet surveys
Source: BMC Infect Dis. 2016 Mar 15;16:127. doi: 10.1186/s12879-016-1442-1 (PMC4793755; doi:10.1186/s12879-016-1442-1)
Supplement: Additional file 1: — Overview questionnaire items. (DOCX 18 kb) [file 12879_2016_1442_MOESM1_ESM.docx]

**Appendix 1 Overview questionnaire items**

**Background characteristics**

- Sex^#^
- Age^#^
- Organisation *(professionals only)*
- Profession *(professionals only)* ^#^
- Education level *(parents only)* ^#^
- Ethnicity *(parents only)*
- Household income *(parents only)*
- Household size *(parents only)*
- Number of children
- Participation of child(ren) in NIP
- Change in opinion on vaccination
- Influence on opinion of vaccination

**Attitude towards universal varicella vaccination** *(professionals only)* ^#^

*Choose between ‘nobody’, ‘risk groups (high risk of severe course)’, ‘all infants’ and/or ‘all susceptible 9-year-olds’:*

- Who is eligible for vaccination against varicella according to you? *(multiple answers possible)*

(🡪 outcome variable for logistic regression analysis professionals)

**Intention regarding vaccination of own child against varicella within the NIP** *(parents only)* ^#^

*Choose between 1=yes, definitely, 2=probably yes, 3=neutral, 4=probably not or 5=no, never:*

- Would you vaccinate your own child(ren) against varicella if the vaccine is included in the NIP?
  (all vaccinations within the NIP are free of charge).

(🡪 outcome variable for logistic regression analysis parents)

**Knowledge about varicella zoster virus (VZV)**

*For each of the statements choose between ‘right’, ‘wrong’ or ‘don’t know’:*

- If you never have had varicella, you can not get herpes zoster^*^
- If the blisters have dried up, you are no longer infectious^*^
- In general, you will get varicella only once in your life^*^

*For each of the questions estimate the occurrence by choosing the correct answer out of 4 different options (see Table 2):*

- What percentage of the Dutch population has had varicella before the age of 12 years? ^*^
- How many people visit their general practitioner for varicella in the Netherlands each year?
- How many people are being hospitalised for varicella or its complications in the Netherlands each year?
- How many people die due to varicella or its complications in the Netherlands each year? ^*^

Knowledge score VZV^#^: sum of the 5 items above with a ^*^ where each correct answer was awarded with 1 point, a wrong or missing answer with 0 points. We did not include the two other knowledge items because this kind of detailed information is logically not expected to be well known by parents in particular.

**Beliefs about the disease varicella and varicella vaccination**

*Please rank the seriousness of the following diseases:*

- Tetanus
- Poliomyelitis
- Pertussis
- Measles
- Mumps
- Rubella
- Pneumococcal disease
- Meningococcal disease
- Varicella.

*Choose between 1=strongly disagree, 2=disagree, 3=neutral, 4=agree or 5=strongly agree:*

- Varicella generally has a mild disease course in healthy children^#^ *(perceived severity)*
- Varicella can cause serious complications^#^ *(perceived severity)*
- Varicella is a disease one could better have been through^#^ *(belief natural experience disease)*
- I am worried about the side effects of varicella vaccination^#^ *(perceived severity side effects of vaccination)*
- I think varicella is a disease serious enough to vaccinate against^#^ *(perceived severity)*
- I think most parents will vaccinate their child against varicella^#^ *(subjective norm)*
- There is no use in keeping children with varicella away from school or child care *(belief transmission)*

**Implementation of varicella vaccination** *(professionals only)*

*Choose between 1=strongly disagree, 2=disagree, 3=neutral, 4=agree or 5=strongly agree:*

- I think a parent should have the possibility to choose between a MMR and a MMRV vaccine for themselves

*Choose between 1=strongly disagree, 2=disagree, 3=neutral, 4=agree or 5=strongly agree:*

- Do you feel able to convince parents of the importance of vaccination against varicella?
- Do you have the intention to advise parents to vaccinate their children against varicella if a vaccine becomes available?
- Do you expect many questions from parents about varicella vaccination? *(CHC professionals only)*
- Would you find it difficult to discuss with parents about varicella vaccination? *(CHC professionals only)*

**Implementation of varicella vaccination** *(parents only)*

*Choose between 1=very bad idea, 2=bad idea, 3=neutral, 4=good idea or 5=very good idea:*

- What do you think about merging varicella with MMR vaccination within one vaccine, so you can no longer choose yourself whether you want a vaccination with or without varicella?

^#^ Included in logistic regression analyses
